# The Impact of BK Channels on Cellular Excitability Depends on their Subcellular Location

**DOI:** 10.3389/fncel.2016.00206

**Published:** 2016-08-31

**Authors:** Tobias Bock, Greg J. Stuart

**Affiliations:** Eccles Institute of Neuroscience and Australian Research Council Centre of Excellence for Integrative Brain Function, John Curtin School of Medical Research, Australian National UniversityCanberra, ACT, Australia

**Keywords:** BK channel, pyramidal neuron, cortex, dendrite, calcium channel

## Abstract

Large conductance calcium-activated potassium channels (or BK channels) fulfil a multitude of roles in the central nervous system. At the soma of many neuronal cell types they control the speed of action potential (AP) repolarization and therefore they can have an impact on neuronal excitability. Due to their presence in nerve terminals they also regulate transmitter release. BK channels have also been shown to be present in the dendrites of some neurons where they can regulate the magnitude and duration of dendritic spikes. Here, we investigate the impact of modulating the activation of BK channels at different locations on the cellular excitability of cortical layer 5 pyramidal neurons. We find that while somatic BK channels help to repolarize APs at the soma and mediate the fast after-hyperpolarization, dendritic BK channels are responsible for repolarization of dendritic calcium spikes and thereby regulate somatic AP burst firing. We found no evidence for a role of dendritic BK channels in the regulation of backpropagating AP amplitude or duration. These experiments highlight the diverse roles of BK channels in regulating neuronal excitability and indicate that their functional impact depends on their subcellular location.

## Introduction

Layer 5 (L5) pyramidal neurons are the primary output neurons in the cortex and therefore play a key role in cortical signal processing. The pattern of action potential (AP) output of these neurons depends in part on the location of incoming synaptic inputs, which are distributed throughout their extensive dendritic tree. If synaptic potentials integrate passively, inputs to the distal apical dendrites of L5 pyramidal cells would not be expected to significantly impact on the somatic membrane potential and therefore on AP output (Stuart and Spruston, 1998; Williams and Stuart, 2002). However, due to a separate calcium spike initiation zone in the apical dendrites L5 pyramidal neurons are effectively compartmentalized into two distinct regions. The basal-somatic region, which mainly receives thalamo-coritcal bottom-up inputs that have a rather direct impact on AP output, and a distal apical region that receives mostly top-down inputs from other cortical areas and generates dendritic calcium spikes promoting AP burst firing at the soma (Schiller et al., 1997; Larkum et al., 1999a,b; Williams and Stuart, 2002). These two regions of L5 pyramidal neurons do not integrate signals independently from each other. Somatic APs actively propagate into the apical dendrites of L5 neurons (Stuart and Sakmann, 1994) and the membrane potential at proximal apical dendritic locations determines the efficacy of forward propagation of dendritic spikes to the soma, and thereby their impact on AP output (Larkum et al., 2001).

Large-conductance calcium-activated potassium channels (BK channels) in the dendrites of L5 pyramidal neurons would be expected to play an important role in regulating the capacity of dendritic spikes to influence AP output. Dendritic calcium spikes are associated with large increases in intracellular calcium as well as significant changes in membrane voltage (Schiller et al., 1997), both of which have a powerful influence on the open probability of BK channels (Latorre et al., 1989). Somatic APs are known to activate BK channels leading to the outflow of potassium and hyperpolarization. As BK channels are fast activating (on the order of 1 ms or less) their activation at the soma speeds AP repolarization and leads to the generation of the fast after-hyperpolarization (Lancaster and Nicoll, 1987; Storm, 1987; Shao et al., 1999; Faber and Sah, 2002). While activation of a potassium channel would normally be expected to have a negative impact on neuronal excitability, by enhancing repolarization of somatic APs and thereby reducing sodium channel inactivation, as well as the activation of inward-rectifier potassium channels, somatic BK channels can increase neuronal firing frequency and therefore excitability (Gu et al., 2007). The role of BK channels on dendritic excitability is less well understood. Dendritic calcium spikes are regenerative electrical events that depend on the activation of dendritic voltage-dependent calcium channels. The effectiveness with which they spread to the soma and influence AP generation will in part depend on their amplitude and duration. Activation of dendritic BK channels would be expected to speed repolarization of the membrane potential during dendritic calcium spikes and therefore decrease the amount of depolarization these spikes generate at the site of AP initiation. Consistent with this idea, previous work indicates that BK channels can curtail dendritic calcium spikes in pyramidal neurons (Golding et al., 1999; Benhassine and Berger, 2009) as well as in cerebellar Purkinje cells (Cavelier et al., 2002; Womack and Khodakhah, 2004), changing the dynamics of AP firing in these neurons.

One caveat with this previous work is that the impact of BK channels on dendritic excitability has only been investigated using bath applications of BK channel blockers, which block both somatic and dendritic BK channels. Blocking somatic BK channels will increase the duration of somatic APs, which could influence the amplitude or duration of backpropagating APs (bAPs; Shao et al., 1999). Blocking somatic BK channels may also influence the refractory period for somatic AP firing (Gu et al., 2007), and thereby the frequency of AP bursts. Both of these effects could have an impact on dendritic excitability. To directly test the role of BK channels at different locations on the regulation of neuronal excitability, we locally applied BK blockers to somatic and dendritic compartments allowing us to investigate the impact of BK channels at different locations in isolation. These experiments showed that in L5 pyramidal neurons somatic/axonal BK channels regulate AP repolarization, whereas dendritic BK channels regulate the duration of dendritic calcium spikes, indicating that the functional impact of BK channels depends on their subcellular location.

## Materials and Methods

### Animal Preparation

Wistar rats (4–6 weeks old) were anesthetized by inhalation of isoflurane (2%) and decapitated according to the procedures approved by the Animal Ethics Committee of the Australian National University. The skull was opened and the brain removed and immediately transferred into ice-cold carbogenated artificial cerebrospinal fluid (ACSF; 125 mM NaCl, 2 mM CaCl_2,_ 1 mM MgCl_2_, 25 mM NaHCO_3_, 3 mM KCl and 1.25 mM NaH_2_PO_4_). During slicing the MgCl_2_ concentration was increased to 5 mM to reduce cell excitability. The brain was cut along the midline and adhered to an angled (10–15°) slicing platform. Using a vibrating tissue slicer (Campden, UK or Leica Microsystems, Germany) 300 μm thick brain slices were cut and transferred to a chamber containing ACSF at 35°C, where they were incubated for 40 min. Thereafter, they were held at room temperature (21°C) until required.

### Electrophysiology

Recordings were made from visually identified L5 pyramidal neurons in primary somatosensory cortex using an Olympus BX61 WI microscope, equipped with Dodt gradient contrast optics (Luigs and Neumann, Germany) and a fluorescent imaging system. In those cases where we recovered the morphological data of recorded cells their morphology was consistent with that of thick tufted L5b neurons. During recording slices were continuously perfused with carbogenated ACSF at a rate of 2 ml/min at 34°C (±1°C). Borosilicate glass pipettes (inner diameter 0.5 mm, outer diameter 1 mm) were pulled by a computer-controlled electrode puller (Sutter Instruments, Novato, CA, USA) and had open tip resistances of 4–6 MΩ for somatic recording pipettes and 9–12 MΩ for dendritic recording pipettes. Recording pipettes were filled with intracellular solution of the following composition: 130 mM K-Gluconate, 10 mM KCl, 10 mM HEPES, 4 mM Mg-ATP, 0.3 mM Na_2_-GTP, 10 mM Na_2_-Phosphocreatine (pH set to 7.25 with KOH and osmolarity 285 mosmol/l). Patch pipettes were electrically connected via a chlorided silver wire to voltage and current-clamp amplifiers via the amplifier headstage, which was mounted on a remotely-controlled micromanipulator (Luigs and Neumann, Germany). All recordings were obtained with BVC-700A amplifiers (Dagan Corp., Minneapolis, MN, USA).

Dual somatic and dendritic whole-cell patch-clamp recordings were established with the dendritic recording site usually 600–800 μm from the soma and not more then 100 μm from the first bifurcation of the apical dendrite. Somatic whole-cell patch-clamp recording was first established and the cell was filled with the fluorescent dye Alexa 594 (5 μM; Molecular Probes, Eugine, OR, USA), which was added to the intracellular solution of the somatic patch pipette. After allowing time for the dye to perfuse into the cell a fluorescent image of the dendritic tree was overlaid on top of the Dodt gradient contrast image using customized software (dancam) to coordinate a pair of shutters (Sutter Instruments, Novato, CA, USA), located prior to the transmitted and epifluorescent light paths. All images were acquired with a CoolSNAP EZ CCD camera (Photometrics, Tucson, AZ, USA). During the experiment, the electrode position was adjusted to maintain proximity to the dendrite if drift in the tissue or pipette was observed. In some cases, slight positive pressure was applied (not more than 3 mmHg) in order to prevent clogging of the pipette tip. Cells were excluded from data analysis if the somatic resting membrane potential was depolarized more than −55 mV, if fluctuations in membrane potential greater than 5 mV were observed at any time during the recording, or if the series resistance exceeded 20 MΩ at the soma or 40 MΩ at the dendritic recording location, or changed by more than 15% during the recording.

### Pharmacology

Drugs were either applied via the bath solution or locally using a glass pipette positioned on top of the slice. The tip of this application pipette was broken under visual control to obtain a diameter of 20–30 μm. This pipette was positioned just above the surface of the slice and drugs were locally applied using 20–30 mmHg of positive pressure. The area of the slice perfused during local application of drugs was estimated to be approximately 150 μm across based on the spread of the fluorescent dye Alexa 594. Applying ACSF to the slice using this method caused no change in the response of the cell during somatic and dendritic current injections, indicating that drug delivery does not cause artifacts due to tissue movement or other unintended side effects. Stock solutions of the BK channel blockers iberiotoxin (IbTX; Tocris, UK) and paxilline (Tocris, UK) were dissolved in purified water (Millipore Systems, Billerica, MA, USA) at a concentration of 100 μM, kept at −20°C and diluted in external solution directly before use. During bath application IbTX and paxilline were applied at concentrations of 100 nM and 1 μM, respectively. Effects were observed 2–4 min after the onset of bath application and data after 10 min were used for analysis. IbTX was locally applied at a concentration of 1 μM, and effects were observed within 30 s to 1 min after application onset. Data 2 min after the onset of local application of IbTx was used for analysis.

### Data Acquisition and Analysis

Electrophysiological data were filtered at 10 kHz and acquired at 50 kHz by a Macintosh computer running Axograph X acquisition software (Axograph Scientific, Sydney, NSW, Australia) using an ITC-18 interface (Instrutech/HEKA, Germany). Data analysis was performed using Axograph X in combination with custom programs in MATLAB (Mathworks, Natick, MA, USA) as well as Microsoft Excel (Microsoft Corp., Redmond, WA, USA). Prism 4 (Graphpad, San Diego, CA, USA) was used for statistics and preparation of graphs. For paired data Wilcoxon’s non-parametric matched pairs test or a paired *t*-test (if a Gaussian distribution could be assumed) was used to test statistical significance. For multiple data sets Dunn’s Multiple Comparison test was performed to determine statistical significance. In the figures a single asterisk (*) indicates a *P* value of <0.05, double asterisks (**) indicates a *P* value of < 0.01, and “NS” indicates “not statistically significant” (*P* > 0.05). Results are presented as average values ± standard error (SE).

## Results

### Impact of Global BK Channel Block

**Figure 1:**
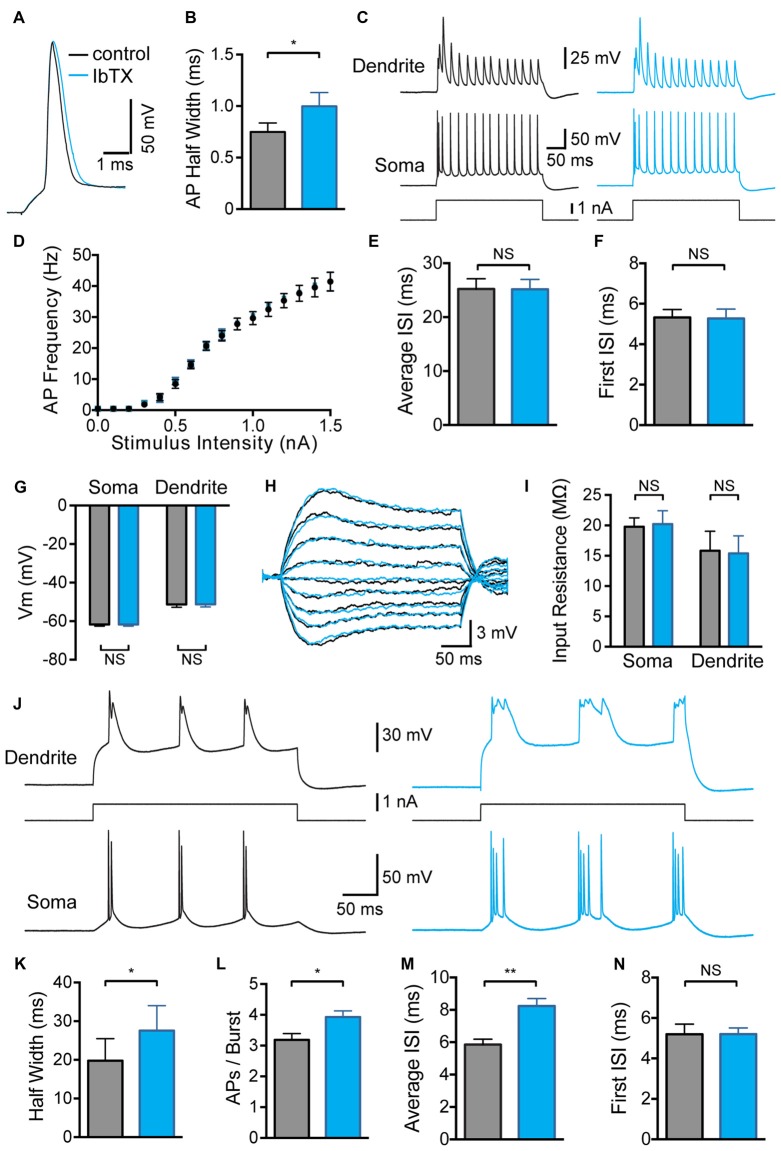
**Effect of global large conductance calcium-activated potassium channels (BK channel) block on active and passive properties. (A)** Single somatic action potential (AP) before (black) and after (blue) bath application of Iberiotoxin (IbTX). **(B)** Average half width of somatic APs before (black) and after (blue) block of BK channels by bath application of IbTX (*n* = 7). **(C)** Somatic and dendritic responses (660 μm from the soma) to somatic current injection before (black) and after (blue) bath application of IbTX. **(D)** Average F-I curve before (black) and after (blue) bath application of IbTX (*n* = 7). **(E,F)** Average inter-spike interval (ISI) between all somatic APs **(E)** or just the first two somatic APs **(F)** during a somatic current step injection of 1.5 nA before (black) and after (blue) bath application of IbTX (*n* = 7 cells). **(G)** Average resting membrane potential at the soma and dendrite recording site (600–800 μm from soma) before (black) and after (blue) bath application of IbTX. **(H)** Responses to sub-threshold current injections (−0.4 to +0.4 nA in steps of 0.1 nA) into the soma of an layer 5 (L5) pyramidal neuron before (black) and after (blue) bath application of IbTX. **(I)** Average input resistance at the soma and dendrite recording site (600–800 μm from soma) before (black) and after (blue) IbTX application (*n* = 7 cells). **(J)** Somatic and dendritic responses (660 μm from the soma) to dendritic current injection before (black) and after (blue) bath application of IbTX. **(K)** Average half width of dendritic calcium spikes before (black) and after (blue) block of BK channels by bath application of IbTX (dendritic recording site 600–800 μm from soma; *n* = 7). **(L)** Average number of APs per burst before (black) and after (blue) bath application of IbTX (*n* = 7). **(M,N)** Average ISI between all somatic APs **(M)** or just the first two somatic APs **(N)** during AP bursts evoked by dendritic current injections of 1.2 nA before (black) and after (blue) bath application of IbTX (*n* = 7 cells).

Bath application of BK channel antagonists was used to assess the impact of global BK channel block on the somatic and dendritic excitability of L5 pyramidal neurons. Consistent with previous studies in pyramidal neurons from the hippocampus (Lancaster and Nicoll, 1987) and amygdala (Faber and Sah, 2002), as well as cortical L5 pyramidal neurons (Benhassine and Berger, 2009), bath application of the BK channel blocker IbTx (100 nM) significantly increased somatic AP half width (Figures 1A,B; *p* < 0.05; *n* = 7). Despite this increase in AP width, we did not observe any impact of globally blocking BK channels on AP firing frequency during somatic current injections, even at the highest stimulus intensities tested (Figures 1C,D; *n* = 7). In addition, there was no change in the average inter-spike interval (ISI) between somatic APs (Figure 1E; *n* = 7) or the ISI between the first two APs evoked by somatic current injections (Figure 1F; *n* = 7). Global BK channel block also had no impact on somatic or dendritic sub-threshold membrane properties. The somatic and dendritic resting membrane potential measured 600–800 μm from the soma were unaffected by bath application of IbTX (Figure 1G; *n* = 7). Bath application of IbTX also had no significant impact on somatic or dendritic input resistance (Figures 1H,I; *n* = 7 cells).

In contrast, global block of BK channels increased the average half width of dendritic calcium spikes evoked by current injection through the dendritic recording pipette (Figures 1J,K; *p* < 0.05; *n* = 7), resulting in an increase in the number of APs evoked during AP burst firing (Figure 1L; *p* < 0.05; *n* = 7). The generation of extra APs at longer delays during bursts led to an increase in the average ISI during dendritic current injections after BK channel block (Figure 1M; *p* < 0.01; *n* = 7). When this analysis was restricted to the first two APs in a burst no change in ISI was detected (Figure 1N; *p* > 0.05; *n* = 7). These data indicate that BK channels in the soma and apical dendrites of L5 pyramidal neurons are activated when the neuron reaches threshold for generation of somatic APs or dendritic spikes, and are closed at sub-threshold membrane potentials. In addition, these data show that globally blocking BK channels increased the duration of dendritic calcium spikes and the strength of somatic burst firing.

### Impact of BK Channel Block on Backpropagating APs

Contrary to a previous study (Benhassine and Berger, 2009), we did not observe any impact of global BK channel block on the amplitude of backpropagating APs (bAPs) measured at distal dendritic locations (Figures 2A,B; 600–800 μm from the soma; *p* > 0.05; *n* = 7). The bAP half width also showed no significant change after wash-in of IbTX (Figure 2C; *p* > 0.05; *n* = 7). Despite no change in average bAP amplitude or duration it is possible that BK channels could modulate bAPs at specific dendritic locations. To investigate this possibility we plotted the bAP amplitude against the distance of the dendritic recording site from the soma for each cell before and after global BK channel block (Figure 2D; *n* = 7). This analysis did not reveal a distance-dependent impact of global BK channel block on bAP amplitude at the dendritic recording locations examined (600–800 μm from the soma).

**Figure 2:**
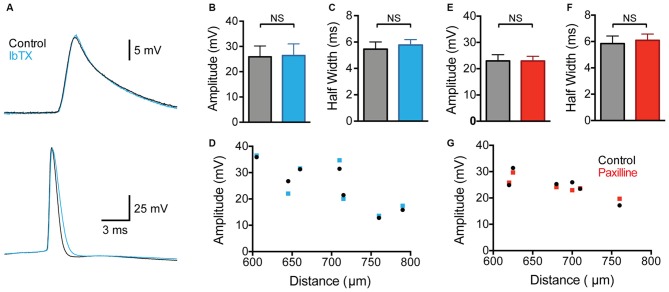
**Effect of global BK channel block on backpropagating APs (bAPs). (A)** Single somatic (bottom) and dendritic (top; 705 μm from the soma) AP evoked by somatic current injection before (black) and after (blue) bath application of IbTX. **(B,C)** bAP amplitude **(B)** and half width **(C)** before (black) and after (blue) bath application of IbTX (*n* = 7). **(D)** bAP amplitude plotted against the distance of the dendritic recording site from the soma before (black) and after (blue) bath application of IbTX (*n* = 7). **(E,F)** Average bAP amplitude **(E)** and half width **(F)** before (black) and after (red) bath application of paxilline (*n* = 6). **(G)** bAP amplitude plotted against the distance of the dendritic recording site from the soma before (black) and after (red) bath application of paxilline (*n* = 6).

As the previous work by Benhassine and Berger (2009) investigated the impact of BK channels on bAPs using the alternative BK channel blocker paxilline, we repeated these experiments using bath application of paxilline (1 μM). These experiments gave essentially identical results to those using IbTX, with no change in average bAP amplitude or half width observed (Figures 2E,F; *p* > 0.05; *n* = 6). In addition, no distance-dependent effects of bath application of paxilline in bAP amplitude were observed at the dendritic recording locations examined (Figure 2G; *n* = 6). In conclusion, these data, collected from a total of 13 cells using two different BK channel blockers, suggest that BK channels do not play a significant role in regulating the amplitude or duration of bAPs in the apical dendrites of cortical L5 pyramidal neurons. The absence of an impact of BK channels on bAPs is consistent with earlier work in hippocampal CA1 pyramidal neurons (Poolos and Johnston, 1999).

### Impact of Local BK Channel Block

**Figure 3:**
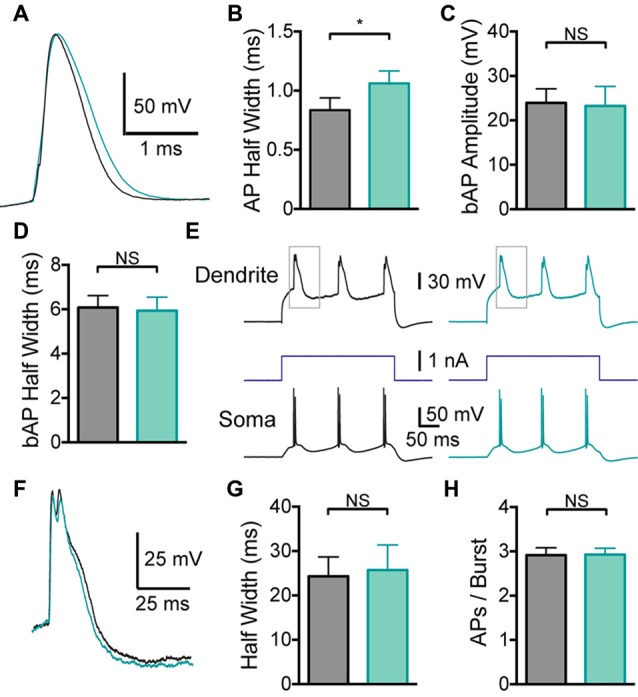
**Effect of somatic BK channels on neuronal excitability. (A)** Single somatic AP before (black) and after (light green) local IbTX application to the soma. **(B)** Average half width of somatic APs before (black) and after (light green) local IbTX application to the soma (*n* = 7). **(C,D)** Average amplitude **(C)** and half width **(D)** of bAPs before (black) and after (light green) local IbTX application to the soma (*n* = 7). **(E)** Somatic and dendritic responses during dendritic current injection before (black) and after (light green) local IbTX application to the soma (dendritic recording site 610 μm from soma). Gray boxes indicate the part of the trace shown in **(F)**. **(F)** Overlay of dendritic calcium spikes recorded before (black) and after (light green) local IbTX application to the soma (aligned at threshold). **(G)** Average half width of dendritic calcium spikes before (black) and after (light green) local IbTX application to the soma (*n* = 7). **(H)** Average number of APs per burst before (gray) and after (light green) local IbTX application to the soma (*n* = 7).

Given that global BK channel block has different effects on somatic and dendritic excitability, which could interact, to determine the impact of somatic and dendritic BK channels in isolation BK channels were blocked locally by applying IbTX to the soma or the dendritic recording site. Somatic application of IbTX caused an increase in somatic AP half width (Figures 3A,B; *p* < 0.05; *n* = 7), but no change in bAP amplitude or width (Figures 3C,D; *p* > 0.05; *n* = 7), as observed during bath applications of IbTX (Figure 2). Somatic applications of IbTX also had no significant impact on the duration of dendritic calcium spikes during dendritic current injection (Figures 3E–G; *n* = 7) or the number of APs generated during burst firing (Figure 3H; *p* > 0.05; *n* = 7).

**Figure 4:**
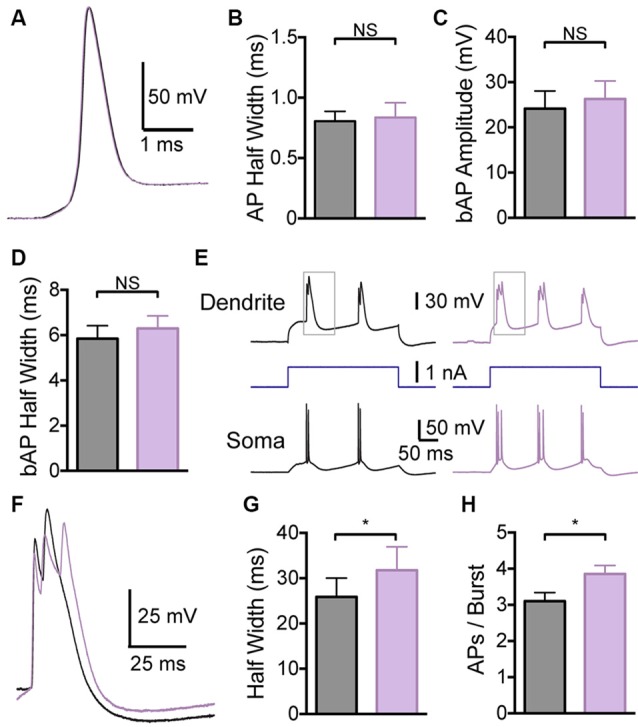
**Effect of dendritic BK channels on neuronal excitability. (A)** Single somatic AP before (black) and after (purple) local IbTX application to the dendritic recording site (640 μm from the soma). **(B)** Average half width of somatic APs before (black) and after (purple) local IbTX application to the dendritic recording site (*n* = 7). **(C,D)** Average amplitude **(C)** and half width **(D)** of bAPs before (black) and after (purple) local IbTX application to the dendrite recording site (600–750 μm from the soma; *n* = 7). **(E)** Somatic and dendritic responses during dendritic current injection before (black) and after (purple) local IbTX application to the dendritic recording site (640 μm from soma). Gray boxes indicate the part of the traces shown in **(F)**. **(F)** Overlay of dendritic calcium spikes recorded before (black) and after (purple) local IbTX application to the dendritic recording site (aligned at threshold). **(G)** Average half width of dendritic calcium spikes before (black) and after (purple) local IbTX application to the dendritic recording site (600–750 μm from the soma; *n* = 7). **(H)** Average number of APs per burst before (black) and after (purple) local IbTX application to the dendritic recording site (600–750 μm from the soma; *n* = 7).

In contrast, local application of the BK channel blocker IbTX to the distal dendritic recording site had no effect on somatic APs (Figures 4A,B; *p* > 0.05; *n* = 7), indicating that these dendritic applications did not spread to the soma. Consistent with observations during bath applications of IbTX, dendritic applications of IbTX had no effect on bAP amplitude or half width (Figures 4C,D; *p* > 0.05; *n* = 7), but increased the half width of dendritic calcium spikes (Figures 4E–G) and also increased the number of APs evoked during subsequent burst firing (Figure 4H; *p* > 0.05; *n* = 7).

## Discussion

The results presented here show that somatic and dendritic BK channels play different roles in regulating the excitability of cortical L5 pyramidal neurons. By locally blocking BK channels we show that somatic BK channels regulate AP duration, whereas dendritic BK channels regulate the duration of dendritic calcium spikes and thereby AP burst firing. No effect of local or global BK channel block on bAPs was observed. These experiments highlight the diverse roles BK channels play in regulating neuronal excitability and indicate that their functional impact depends on their subcellular location.

BK channels had no impact on the sub-threshold membrane potential in L5 neurons. This is presumably due to the fact that activation of BK channels requires significant increases in intracellular calcium concentration and membrane depolarization (Storm, 1987). These conditions are only met at the soma when the cell is firing somatic APs or in the dendrites during generation of dendritic calcium spikes. A previous study by Benhassine and Berger (2009) suggested that BK channels also act to dampen bAP amplitude and duration at distal dendritic locations in cortical L5 pyramidal neurons. We observed no evidence for this in our experiments (Figure 2). The reason for this discrepancy with the earlier work is unclear; however, the conclusions of Benhassine and Berger (2009) were based on a small sample size (three recordings at locations greater than 400 μm from the soma) and were not quantified. Our finding that bAPs in the apical dendrites of L5 pyramidal neurons are not regulated by BK channels is based on a much larger dataset (13 recordings; 600–800 μm from the soma) and is consistent with earlier work in hippocampal CA1 pyramidal neurons (Poolos and Johnston, 1999). The finding that bAPs are not regulated by BK channels in both cortical L5 and hippocampal CA1 pyramidal neurons presumably indicates that the dendritic depolarization and/or calcium influx associated with bAPs in these neurons is insufficient to drive BK channel activation. While single bAPs do not cause BK channel activation in the distal dendrites of L5 pyramidal neurons, trains of somatic APs at high frequencies can trigger dendritic calcium spikes (Larkum et al., 1999a). During high frequency AP trains BK channels can be activated where they act to dampen dendritic calcium influx (Benhassine and Berger, 2009; Grewe et al., 2010).

In hippocampal CA1 pyramidal neurons the enhanced repolarization of somatic APs by BK channels can increase AP output at high firing frequencies (Gu et al., 2007). Modeling suggests that this occurs through a reduction in inactivation of voltage-gated Na^+^ channels (Gu et al., 2007), which effectively reduces the refractory period between APs. In contrast, in L5 pyramidal neurons blocking BK channels did not affect the number of APs evoked by somatic current injections or the ISI even at the highest current amplitudes tested (Figures 1C–F). In addition, we did not observe an impact of blocking BK channels on the first ISI during AP bursts evoked by dendritic current injections (Figure 1N), although the average ISI was increased (Figure 1M). This increase in average ISI during dendritic current injections was due to the generation of additional somatic APs at later times during AP bursts (Figure 1J). We conclude that BK channels do not impact on the AP refractory period of L5 pyramidal neurons. These data suggest that somatic/axonal BK channels in L5 neurons do not have a direct effect on AP firing, and presumably serve other functions, such as regulating intracellular calcium levels. In contrast, BK channels located in the apical dendrite control the number of APs evoked during AP bursts.

The most notable effect of blocking dendritic BK channels was the increase in the width of dendritic calcium spikes, which is consistent with the results of Benhassine and Berger (2009). BK channels are expressed along the length of the apical dendrites of L5 pyramidal neurons (Benhassine and Berger, 2005), as are high-threshold voltage-gated calcium channels (Westenbroek et al., 1992; Kelly et al., 2001). These calcium channels are opened during dendritic calcium spikes (Schiller et al., 1997; Pérez-Garci et al., 2013) and presumably provide the increase in intracellular calcium needed to activate BK channels. Once activated, K^+^ outflow through BK channels repolarizes the membrane potential and thereby reduces the duration of dendritic calcium spikes. An impact of BK channels on dendritic calcium spikes is not unique to pyramidal neurons in the cortex and has also been observed in cerebellar Purkinje cells (Cavelier et al., 2002; Womack and Khodakhah, 2004; Rancz and Häusser, 2006) and hippocampal pyramidal neurons (Golding et al., 1999). In contrast, as mentioned above, bAP amplitude and half-width were not affected by BK channel block, as is also the case in hippocampal pyramidal neurons (Poolos and Johnston, 1999). To what extent the findings described here in thick tufted, putative L5b neurons can be extrapolated to other types of cortical pyramidal neurons in somatosensory cortex, or to other brain regions, remains to be determined.

To investigate the impact of somatic compared to dendritic BK channels on neuronal excitability in isolation we locally applied BK blockers to somatic and dendritic locations. Using local applications of BK channel blockers we found that dendritic BK channels alone are responsible for controlling the duration of dendritic calcium spikes in cortical L5 pyramidal neurons (Figure 4), whereas somatic/axonal BK channels are responsible solely for repolarization of somatic APs (Figure 3). These experiments indicate that BK-dependent changes in somatic AP duration or refractory period do not play a role in the regulation of dendritic calcium spikes or associated burst firing in L5 neurons. The differential effect of somatic compared to dendritic BK channels on neuronal excitability provides further evidence for functional compartmentalization in L5 pyramidal neurons, and indicates that the role of BK channels in regulating cellular excitability is determined by their subcellular location. This does not mean, however, that the impact of somatic and dendritic BK channels remains confined to the compartment where they are activated. While blocking somatic BK channels had no impact on dendritic excitability, blocking dendritic BK channels increased the duration of dendritic calcium spikes and promoted AP burst firing at the soma. This local, rather than global, role of BK channels in the regulation of neuronal excitability is enhanced by co-localization of BK channels with calcium channels in micro-domains, where their activation is driven by calcium influx through specific, co-localized calcium channels (Marrion and Tavalin, 1998; Isaacson and Murphy, 2001; Sun et al., 2003; Berkefeld et al., 2006; Indriati et al., 2013).

## Author Contributions

TB and GJS: performed and planned experiments, analyzed and interpreted data and wrote the article.

## Conflict of Interest Statement

The authors declare that the research was conducted in the absence of any commercial or financial relationships that could be construed as a potential conflict of interest.
